# Computational models of liver fibrosis progression for hepatitis C virus chronic infection

**DOI:** 10.1186/1471-2105-15-S8-S5

**Published:** 2014-07-14

**Authors:** James Lara, F Xavier López-Labrador, Fernando González-Candelas, Marina Berenguer, Yury E Khudyakov

**Affiliations:** 1 Division of Viral Hepatitis, Centers for Disease Control and Prevention, Atlanta, GA, USA; 2 Joint Research Unit on Genomics and Health FISABIO-Salud Pública and Universitat de Valencia, Valencia, Spain; 3 CIBEResp, National Network Center for Research on Epidemiology and Public Health, Instituto de Salud Carlos III, Spain; 4 Hepatology-Liver Transplantation Unit, Hospital Universitari La Fe and Universitat de Valencia, Valencia, Spain; 5 CIBERehd, National Network Center for Hepatology and Gastroenterology Research, Instituto de Salud Carlos III, Spain

## Abstract

**Background:**

Chronic infection with hepatitis C virus (HCV) is a risk factor for liver diseases such as fibrosis, cirrhosis and hepatocellular carcinoma. HCV genetic heterogeneity was hypothesized to be associated with severity of liver disease. However, no reliable viral markers predicting disease severity have been identified. Here, we report the utility of sequences from 3 HCV 1b genomic regions, Core, NS3 and NS5b, to identify viral genetic markers associated with fast and slow rate of fibrosis progression (RFP) among patients with and without liver transplantation (n = 42).

**Methods:**

A correlation-based feature selection (CFS) method was used to detect and identify RFP-relevant viral markers. Machine-learning techniques, linear projection (LP) and Bayesian Networks (BN), were used to assess and identify associations between the HCV sequences and RFP.

**Results:**

Both clustering of HCV sequences in LP graphs using physicochemical properties of nucleotides and BN analysis using polymorphic sites showed similarities among HCV variants sampled from patients with a similar RFP, while distinct HCV genetic properties were found associated with fast or slow RFP. Several RFP-relevant HCV sites were identified. Computational models parameterized using the identified sites accurately associated HCV strains with RFP in 70/30 split cross-validation (90-95% accuracy) and in validation tests (85-90% accuracy). Validation tests of the models constructed for patients with or without liver transplantation suggest that the RFP-relevant genetic markers identified in the HCV Core, NS3 and NS5b genomic regions may be useful for the prediction of RFP regardless of transplant status of patients.

**Conclusions:**

The apparent strong genetic association to RFP suggests that HCV genetic heterogeneity has a quantifiable effect on severity of liver disease, thus presenting opportunity for developing genetic assays for measuring virulence of HCV strains in clinical and public health settings.

## Background

Hepatitis C virus (HCV) is a major cause of liver disease world-wide and the leading cause of liver transplantation in developed countries [1,2]. There are 7 major genotypes divided into >100 subtypes [3,4], with genotype 1 being responsible for the majority of HCV-infections world-wide [5]. Approximately 70%-80% of HCV-infected patients fail to clear the virus, and develop chronic HCV infections, which is a risk factor for liver diseases such as fibrosis, cirrhosis and hepatocellular carcinoma [6]. Liver fibrosis, which results from an excessive connective tissue built up in the liver (fibrogenesis), can gradually exacerbate during the course of the infection and lead to scarring of the tissue (i.e., cirrhosis) and more severe liver dysfunction. The rate of fibrosis progression (RFP) in HCV infection has been proposed to be classified into 3 categories: fast, intermediate, and slow [7].

A wide array of host factors and conditions has been reported to affect the RFP and predispose patients with chronic HCV infection to rapid progression of liver fibrosis [8]. These include patients with the vitamin D receptor (VDR) bAt|CCA haplotype in combination with low levels of vitamin D [9], reduced expression levels of the transcription factor RelA protein [10], a high body mass index [11,12], elevated ALT levels [12] and older age [7,12,13]. Accelerated RFP has also been associated with male gender, excessive alcohol consumption, age at acquisition of HCV infection [7,11,13] and to immuno-suppression in liver-transplanted patients [14].

Viral factors associated with rapid progression of liver fibrosis have also been identified. Based on data collected from the Swiss Hepatitis C Cohort Study (SCCS) [15], Bochud and colleagues found that among genotypes 1-4, genotype 3 was significantly associated with faster RFP [16]. In liver-transplanted patients, high level of viremia at the time of transplantation has also been found significantly associated with faster RFP [14]. A phylogenetic association of the core sequences with fibrosis scores was observed among HCV strains recovered from post-transplanted patients, suggesting that RFP is a heritable trait [17]. Minimum-spanning tree analysis showed association of 2 HCV genomic regions, core and NS5B, with RFP in non-transplanted patients. However, to date, studies examining the HCV genetic factors as predictors of RFP in patients with chronic HCV infections have been inconclusive [7,13,17-20]. Nonetheless, these observations, taken together with findings indicating associations of genotype with HCV-related steatosis [13,21] and diversity of intra-host HCV variants with liver disease progression [22], suggest that the genetic composition and diversity of HCV strains may affect RFP in patients with chronic HCV infections.

Recently, we have shown that epistatic connectivity among viral genomic sites is strongly associated with host factors such as age, gender and race [23] as well as with interferon or lamivudine resistance [23,24], indicating that intra-host viral evolution is convergent and clinically important traits can be predicted by modeling coevolution among viral genomic sites. Here, we investigated epistatic connectivity among nucleotide sites from 3 regions, core, NS3 and NS5B, of HCV genome and its association with RFP. This is the first study to report the development of computational models with capacity for accurately predicting RFP based on the HCV genetic diversity and composition.

## Methods

### Patients and HCV 1b sequences

HCV 1b consensus nucleotide (nt) sequences of the core, NS3 and NS5b (genome positions: 345-731, 4464-4685 and 8276-8612; spanning polyprotein positions 2-130, 1375-1448, and 2646-2757, respectively) were obtained from GenBank (Accession numbers: AY898811 to AY898940). Sequences of HCV 1b isolates were identified through a study on cohorts of liver transplanted (TOH; n = 22) and non-transplanted - immunocompetent - patients (IC; n = 22) [18]. In order to conduct an interaction/dependency-based analysis, sequences from these three genomic regions were concatenated into a single nt sequence, aligned and annotated with clinical data. Sites were numbered according to their position in the genome using HCV isolate Con1 as reference sequence (GenBank accession number: AJ238799). Sites that were conserved and/or presented an ambiguous nt were removed from the alignment. A total of 174 polymorphic sites were used. Then, data were further reduced to a subset of selected relevant nt sites (n = 25) to conduct analysis of the HCV genetic diversity association to RFP.

Sequence profiles were divided into 2 RFP classes, fast (n = 17) and slow (n = 25), as described in [18]. For purposes of model evaluations, data were also divided by liver transplant status associated to sequence profiles: TOH dataset (n = 22; comprising 10 patients with fast and 12 with slow RFP) and IC dataset (n = 20; comprising 7 patients with fast and 13 with slow RFP). In addition, random-labeled datasets from TOH and IC were also generated in which HCV sequence profiles were randomly assigned to RFP classes. Two patients (IC, fast RFP) were excluded from our analysis as sequences of the HCV NS3 region were not obtained from these patients [18].

### Selection of relevant HCV genetic features

Polymorphic nt sites with the highest degree of correlation to the patients' yearly RFP (fast or slow) were determined using the correlation-based feature subset selection (CFS) method [25], which is based on the "merit" heuristic. The merit heuristic of a feature subset *S *containing *k *features is defined as,

$$Merits=\frac{k\times \bar{rca}}{\sqrt{k+\left(k-1\right)\times \bar{raa}}}$$

where $\bar{rca}$ is the average feature-class correlation and $\bar{raa}$ is the average feature-feature inter-correlation.

CFS iterates through subsets of highly correlated and non-redundant features to find the best subset of interacting features (i.e. features whose values are dependent on the values of other features and the class, and as such, provide additional information about the class). The Best-first greedy search strategy [26] was used in CFS iterations, which considers effects of adding (or removing) a feature to the current subset in order to find a better subset of interacting features. The Best-first search was started with an empty set of features and generated all possible single feature expansions [27]. The subset with the highest merit is chosen and expanded by adding features one at a time. If expanding a subset results in no improvement in the merit score, the search drops back to the next best unexpanded subset and continues from there. The best subset found is returned when search terminates. Here, the Best-first search was performed in the forward direction. The subset of relevant viral features selected on the basis of the Merit heuristic served as the viral parameters to derive models of RFP-specificity of the HCV strains.

### Linear projection graphs and models

To uncover interactions among relevant viral features associated to RFP characteristics of patients and provide information about inter- and intra-RFP class similarities among HCV strains, a linear projection (LP) method [28] was used. This machine-learning method, which takes into consideration interactions among attributes, finds a linear combination of features so that when mapped onto a 2-dimensional (2D) graph the projected data exhibit a trait-specific structure, such as clusters. To find the most useful projections comprising a subset of features (base vectors) that would optimally associate HCV variants to RFP characteristics of patients we used the VizRank search method [29]. Computations were carried out on dataset of relevant features of HCV 1b isolates from 42 patients. To evaluate projections, a scoring function based on the measure of classification accuracy was used, which is common in machine-learning. For each projection, the average probability $\left(\bar{P}\right)$ assignment to the correct RPF-class was computed using a probabilistic k-nearest neighbors algorithm (k-NN), where the parameter k was set at 6. Given enough time, the VizRank search method will explore the entire search space, so it is common to limit the projections' size (subset of features) and/or to limit search times. We limited the global search on dataset to 500 minutes, projections size to up to 11 features and kept the list of projections returned by VizRank to a maximum of 5,000. We then extended the search to the local list of 50000 projections to find optimal projections. This local search was manually terminated after a period of ~4,320 minutes while retaining a list of 20,000 projections (comprised of up to 13 features). Projections with classification scores ≥90.0% were examined and manually selected on the basis of how well they could visually separate HCV strains into RFP-specific clusters.

To generate LP models, the 9-feature-based projection was mapped into LP graph. Then, the FreeViz machine-learning method [28] was used to generate LP models of the projection. This method searches the space for optimal orientations and order placements of base vectors that best represents data classes in the graphs. LP models based on RFP-relevant viral features in selected projections were evaluated on HCV 1b data collected from the 42 patients by 10 repetitions of 70/30 split cross-validation (CV), i.e., randomly sampling 70.0% of data for training and testing classification performance of the model on the remaining 30% of samples. In addition, to validate LP models and viral parameters in selected projections, classification performance of LP models trained on a specific liver-transplant group dataset (see Patients and HCV 1b sequences in Materials and Methods) was measured using the opposite liver-transplant group data as test sets. All analyses related to LP graphs and evaluation of LP models were conducted using the Orange software (v2.0) [30].

Representation of physicochemical properties of HCV nt sequences was achieved by transforming the standard 4-letter alphabetical nt sequence profile of HCV variants into N × 5 dimensional numerical vectors, where N is the sequence length and 5 represents the number of physicochemical values assigned to a nt base, which were based on experimentally measured properties of nt bases (hydrophobicity, polarity, dipole moment, surface area and stacking area) [31].

### Bayesian network classifier (BNC)

To examine how dependency in nt substitutions among relevant genomic sites associate HCV genetic heterogeneity of strains to RFP and to further explore inter- and intra-RFP-class similarities among HCV strains between TOH and IC patients, the learning Bayesian network (BN) approach [32] in the form of a BN classifier (BNC) was used. BN is a probabilistic graphical model, where nodes in the graph represent random variables in data - herein, RFP of host and relevant genetic features of the HCV 1b strains - and arcs in graph represent direct dependencies between the variables. A BNC can represent genomic sequence information and associated metadata in an integrated data-structure (network structure - representing dependencies among features, conditional probability distributions, etc.) to qualitatively and quantitatively assess dependency among genetic features and target features (associated metadata). BNC models can handle problems of convergent evolution when estimating HCV resistance to treatment [23,24] and host-related features, such as, demographic characteristics of patients [23].

Derivation of BNC consists of two tasks, selection of a learned-BNC structure and parameter estimation of BNC. The structure-learning task was conducted in two steps. First we initialized BNC as a naïve BNC, where arcs from the RFP node, representing the yearly RFP characteristics of patients, were directed to each of the nodes representing relevant HCV nt sites. Then, relationships (dependency) among the HCV genetic features were learned from data in an unsupervised fashion using a greedy-search heuristic, the K2 algorithm [33]. For this 2nd step, a constraint in the maximum number of parents (arcs direct towards any given node) was enforced (set to a maximum of 3). The scoring function criterion used in final selection of the BNC structure was based on the Bayesian with Dirichlet priors, BDe metric [34]. The second task, parameter estimation, which consists of estimating the conditional probability tables of nodes in BNC, was directly estimated from data and based on frequency counts.

The BNC model where all features depends on the class and any feature depends on *k *other features at most is described by the formula,

$$p\left(c|f_{1},...,fn\right)\propto p\left(c\right)p\left(f_{1}|f_{i},....,f_{k},c\right)\times ....\times p\left(fn|f_{h},....,f_{k},c\right)$$

where c is the RFP-class and {f_1_, f_i_, f_h_, f_k_, .... fn} are features, CFS-selected sites of the HCV genome used in this study whose values represent nt states (4-letter alphabetical code). The threshold of BNC output boundary between the RFP-classes was set at 0.5.

Two BNC models were generated: the BNC-TOH, learned from TOH cohort HCV 1b isolates and BNC-IC, learned from IC cohort isolates for the purpose of conversely evaluating prediction performances of BNC on test sets from opposite cohort data (tests with unseen data). In order to maintain equal representations of RFP-class examples, size of the IC dataset used to train BNC-IC was reduced from 20 to 14 samples (7 fast and 7 slow fibrosers) by randomly selecting HCV sequences from data.

In addition, to support predictions of models and to account for possible random correlations in data we conducted the same evaluation assessments on BNC models parametized on random-labeled datasets (_Rand_BNC). For these experiments, the structure learning step was skipped. Instead, fixed structure-learning was performed by selecting the BNCs learnt from non-randomized data for the training phases on randomized datasets. A total of 5 randomly-labeled datasets were generated by randomly re-sampling HCV data to conduct 5 repetitions of evaluations of _Rand_ BNC performance on validation tests.

### Evaluation of LP and BNC models

Four metrics were used to evaluate capacity of the models to predict RFP-class: classification accuracy (CA), sensitivity (SN), specificity (SP) and the F-measure,

$$CA=\left[\;\left(TP+TN\right)÷\left[\left(TP+FN\right)+\left(FP+TN\right)\times 100\right]\;\right]$$

$$SN=TP÷\left(TP+FN\right)\times 100$$

$$SP=TN÷\left(FP+TN\right)\times 100$$

$$FMEASURE=\frac{2\times TP}{2\times TP+FP+FN}$$

where TP is the number (No.) of true positives; TN, No. of true negatives; FP, No. of false positives and FN, No. of false negatives.

## Results and discussion

### RFP-specific genetic features of HCV

The heuristic method used to identify which polymorphic nt sites are most relevantly associated to RFP is based on the hypothesis that good features have high correlation to classes and less inter-correlation with each other [35]. This method has been shown to be efficient in finding useful features from data and improves accuracy of machine-learning classifiers [25]. The most RFP-relevant features of HCV are shown in Table 1. Genetic heterogeneity at several nt sites in the HCV Core (n = 8), NS3 (n = 8) and NS5b (n = 9) regions was found to be associated to RFP-class (i.e., fast- and slow-RFP) by CFS analysis. Based on the merit score of the CFS feature subset, it was found that no single nt site or any individual HCV genomic region could strongly and independently explain RFP features. This finding is concordant with a previous study on same HCV 1b data, which demonstrated absence of a RFP-specific clustering in phylogenetic trees of variants of the HCV Core, NS3 and NS5b regions [18]. The relatively low merit score of the CFS feature subset and, consequently, of the feature subsets of individual regions (see legend, Table 1) suggest at least two possible explanations. First, the data contain trivial information grossly unassociated with RFP and, therefore, features in data are useless for the purpose of the RFP classification, which is not likely since the prior analyses showed that sequence data have information related to RFP [18,36]. However, even in such a case, it's been shown that features whose values have low predictive power and appear completely irrelevant, when combined, can still contribute significantly to machine-learning classification [25,37]. Second, the observed merit scores most likely reflect an overall highly complex genetic relationship to RFP phenotype and that CFS-selected HCV genetic features have values predictive of a small area of the RFP space, which may be obvious only under certain conditions (e.g. methods of data analysis, immune status of patient, etc.). In fact, a positive correlation between RFP and genetic diversity of variants of the HCV Core and NS5b regions from non-transplanted and immunocompetent patients was observed by minimum spanning network analyses, however, not in HCV strains from liver transplant recipients [18].

**Table 1:** RFP-relevant HCV sites.

Method	Sites^§^	Score
**CFS^a^**	**450**, 500, 548, 581, 584, 602, 630, 659, **4484**, 4492, **4496**, 4514, 4535, 4538, 4560, 4610, 8324, 8342, **8378**, **8435**, 8438, **8480**, 8540, 8546 and **8606**	**0.35**

**VizRank^b^**		

**12-feature projection**	8480X5, 4496X2, 4484X1, 450X3, 4496X4, 8606X5, 8480X1, 8606X1, 8435X5, 8378X4, 4496X1, 4496X3	**90.38%**

**9-feature projection**	4496X4, 8480X5, 450X3, 8606X4, 4484X4, 8435X5, 8606X3, 4496X5, 8378X2	**90.17%**

^§^Genomic positions assigned based on reference sequence Con1.

^a^A total of 3,757 feature subsets were evaluated by CFS search method. Scoring metric is based on the merit heuristic. Merit scores of feature subsets of the individual regions were also computed: core (0.19), NS3 (0.28) and NS5b (0.20). CFS sites, physicochemical properties of which were selected for the RFP-relevant projections are shown in bold.

^b^Features are listed in the placement order of base vectors in LP graphs shown in Fig. 1. Site-specific physicochemical properties are denoted X1(hydrophobicity), X2(polarity), X3(dipole moment), X4(surface area) and X5(stacking area). See methods for details on scoring metric.

Because integration of features with values of low predictive power or that specify a small fraction of one or more class spaces into a computational framework that combines the values with trait-specific interactions and/or dependencies among features has been effective in identifying HCV markers associated to complex phenotypic traits [23,38], this approach was chosen to resolve the HCV genotype to RFP phenotype association.

### RFP-specific clustering of HCV strains in LP graphs

**Figure 1:**
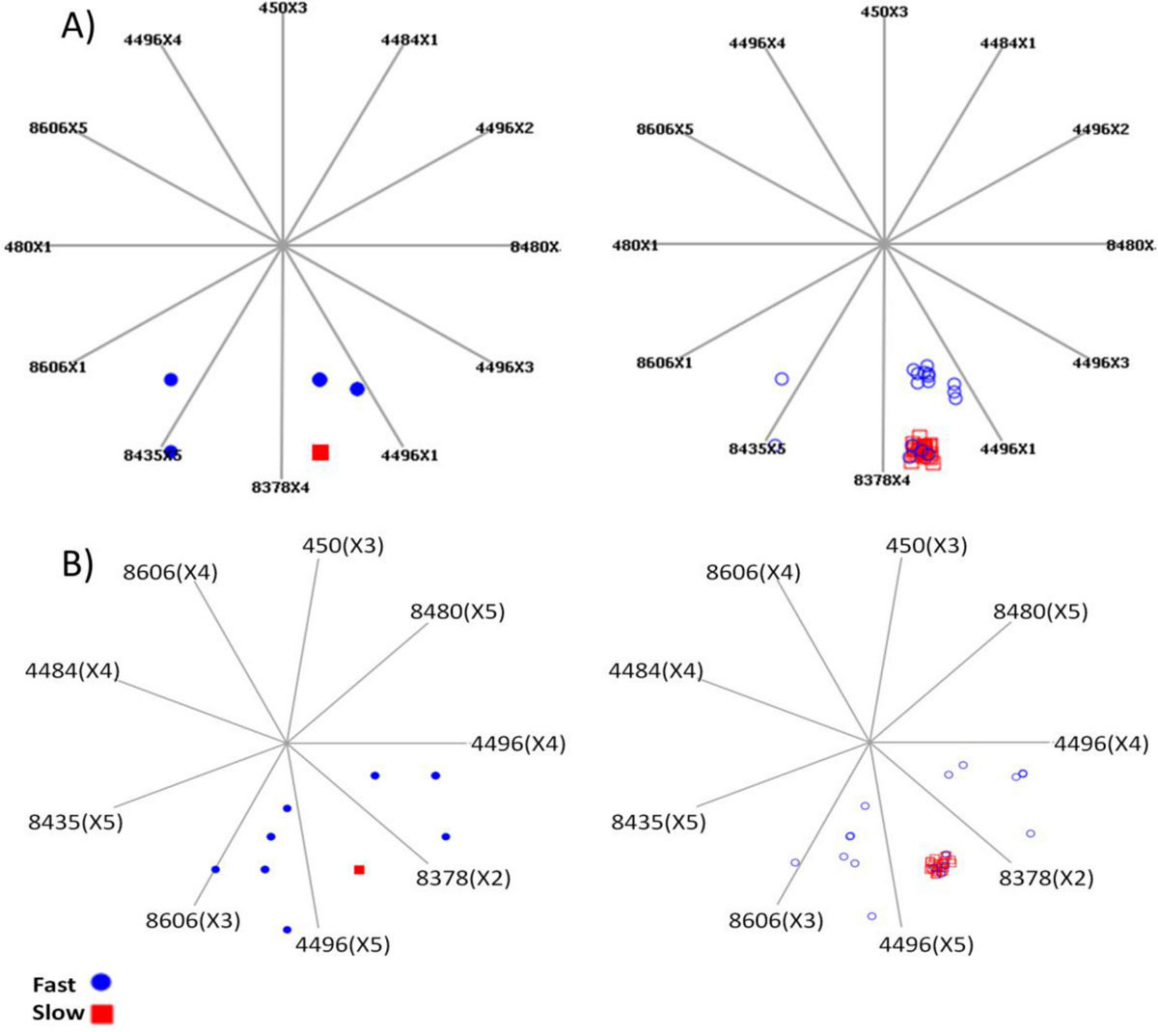
**2D linear projection (LP) graphs**. Base vectors of projection represent RFP-relevant features. Sites are identified by their positions in the HCV genome and physicochemical properties are shown in parenthesis as X1-X5. See Table 1 for detail. LP graphs of HCV 1b isolates (n = 42) sampled from TOH and IC patients based on A) 12-feature and B) 9-feature projections are shown. To the right of each LP graph is the same graph except for the condition that data points were jittered [41] to highlight membership and size of clusters.

A total of 2,911 projections out of the list of 20,000 projections returned by VizRank achieved classification score that ranged between 90.0% and a maximum of 90.42%. Two projections comprising subsets of RFP-relevant nt physicochemical properties of HCV sequences (Table 1) were found to provide the most marked visual division of HCV strains into the RFP classes. LP graphs of the selected RFP-specific projections are shown in Figure 1. Clustering of HCV 1b strains in LP graphs showed strong association to the yearly RFP features of patients, which was projected onto LP graph with as little as 9 physicochemical features from 7 relevant nt sites. It is important to note that the observed clustering in LP graphs corresponds to a true property of the data points because features used to represent nt sequence profiles of HCV strains are continuous values, hence, a consequence of properties ensuing from values assigned to the 4 nt-bases and not a consequence of the visualization [29].

Both the 9- and 12-feature-based LP graphs revealed a tight cluster of HCV strains obtained from IC and TOH patients with slow RFP (n = 25, 13 from IC and 12 from TOH patients). While strains from patients with fast RFP exhibited a broader degree of property variations as they were more dispersed and mostly separated into two spaces in the LP graphs. The apparent RFP-specific clustering among HCV strains in LP graphs together with the level of intermixing observed between IC- and TOH-related strains suggest that RFP may be directly predicted from the HCV sequences irrespective of the transplant status of host.

### LP models of the HCV RFP-specificity

**Figure 2:**
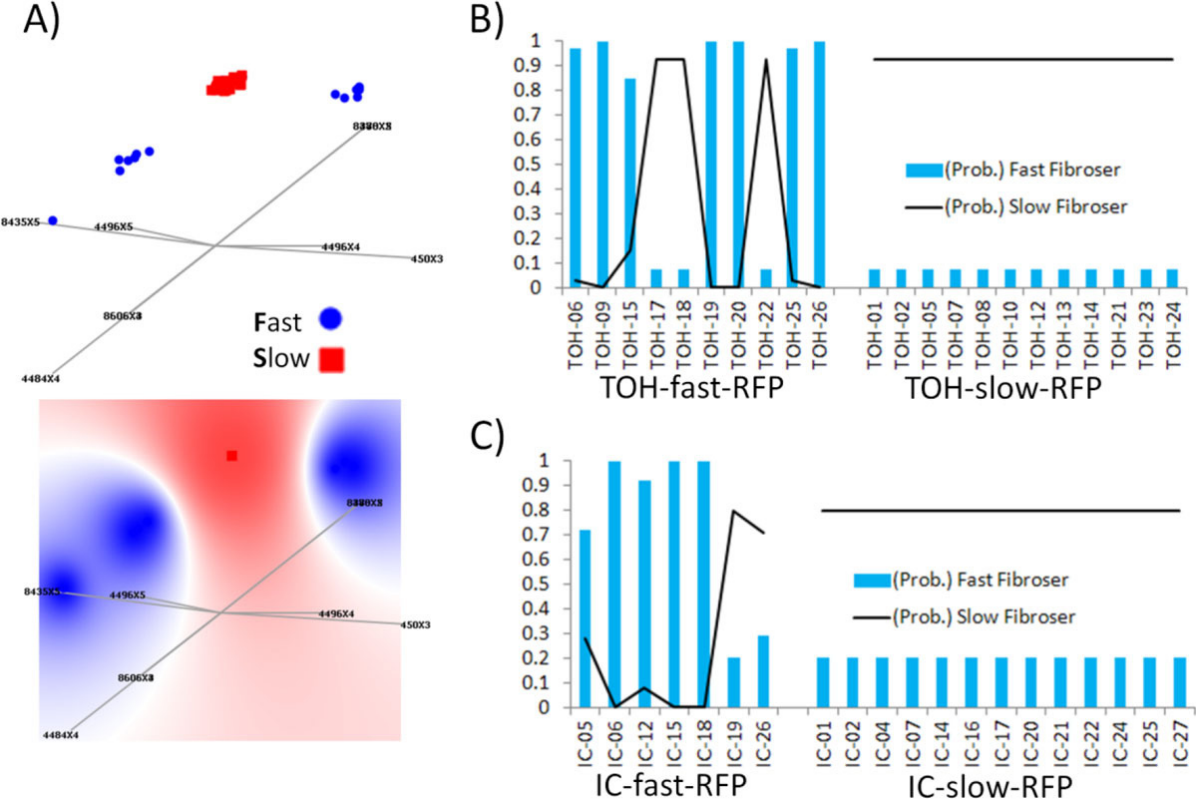
**RFP-specificity of LP models**. A) 2D graph of the LP model shown in Fig 1. HCV strains distributed into 4 clusters, from left to right: cluster 1 (fast RFP-IC, n = 1), cluster 2 (fast RFP-IC, n = 2; fast RFP-TOH, n = 4), cluster 3 (slow RFP-IC, n = 13; slow RFP-TOH, n = 12, and fast RFP-IC, n = 1; fast RFP-TOH, n = 3) and cluster 4 (fast RFP-IC, n = 3; fast RFP-TOH, n = 3). The graph below, shows mapping of computed probability potentials in LP model defining three RFP-class spaces of HCV strains (fast-RFP in blue, slow-RFP in red), where color density of areas are directly proportional to the probability of association to the respective RFP-class. Plots, where x-axis represents predicted probabilities and y-axis denotes observed RFP-class of HCV strains, show classification performances in validation tests of the B) LP-IC and C) LP-TOH model.

An LP model, generated in an automated fashion by FreeViz [28] using the HCV dataset from 42 patients and based on the RFP-relevant 9-feature projections (Table 1) is shown in Figure 2A. The optimally rearranged base vectors in LP model grouped HCV strains into three spaces in the graph, two of which were associated to the fast RFP-class and separated by an area associated to the slow RFP-class. Both fast-RFP associated spaces contained only HCV strains sampled from patients with fast RFP, with strains from IC and TOH patients being evenly distributed between spaces. While the slow-RFP associated space contained 4 HCV strains sampled from patients with fast-RFP. Evaluation of the 70/30-split CV tests showed that the LP model had 93% accuracy of RFP-classification (Table 2). Similarly, accuracy of ≥90.0% was observed for LP-TOH and LP-IC models (models specifically generated from the TOH and IC datasets, respectively; Table 2). Furthermore, only slight decline in classification accuracy was observed in validation tests of LP-TOH and LP-IC, indicating that the models captured general genetic properties associated with RFP from both cohorts of patients and, therefore, are very robust. This finding supports the aforementioned observation in LP graphs that the association between the HCV genetic diversity and RFP is not affected by the transplant status of patients.

**Table 2:** Performance evaluations of models.

Model^‡^	Dataset	CV test^§^	CA (%)	Sensitivity (%)	Specificity (%)	F-measure (%)
LP	Full (n = 42)	70/30-CV	93.20	82.00	100	90.11

LP-TOH	TOH (n = 22)	70/30-CV	90.00	76.67	100	86.79

LP-IC	IC (n = 20)	70/30-CV	95.00	85.00	100	92.31

	**Validation test sets**					

LP-TOH	IC (n = 20)		90.00	71.43	100	83.33

LP-IC	TOH (n = 22)		86.36	70.00	100	82.35

BNC-TOH	IC (n = 20)		85.00	71.43	92.31	82.90

BNC-IC	TOH (n = 22)		86.40	70.00	100	85.62

*_Rand_*BNC-TOH	IC (n = 20)		45.00	na	na	na

*_Rand_*BNC-IC	TOH (n = 22)		45.54	na	na	na

**^‡^**LP model is based on the selected projection comprised of 9 HCV features. Classification accuracy for _Rand_BNCs was averaged over 5 repetitions.

**^§^**Values were averaged for 10 repetitions.

### BNC models of the HCV RFP-specificity

**Figure 3:**
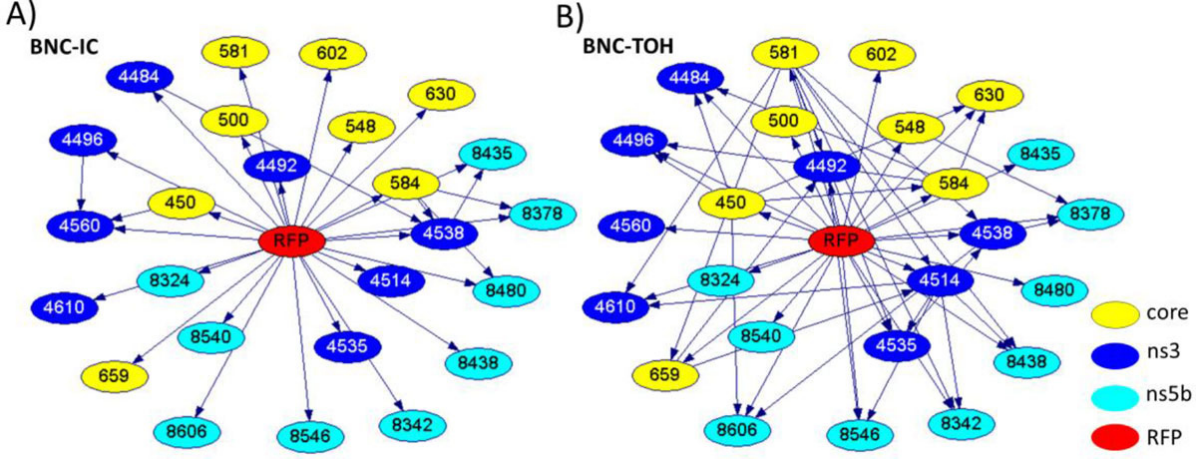
**BNC models of RFP-relevant HCV sites**. Nodes in the graph represent 25 nt sites (Table 1) and arcs between them represent relationships. Numbering of nodes in BNC denotes genomic position in Con1 as reference and colour represent genomic region. Node representing RFP is coloured in red. Models learned from HCV sequence profiles sampled from A) non-transplanted patients and from B) transplanted patients are shown.

Findings from the LP graphs and models indicate that the CFS-selected features can be used as genetic markers of RFP. To further examine these features, a set BNC models was learned from IC and TOH datasets (Figure 3). Network structures were markedly different between BNC models. However, considering the small size of each dataset, it is expected that presence or absence of a single sequence may have a significant effect on the BN structure, thus making unreliable any assertions regarding the specificity of interactions or dependencies among polymorphic nt sites of the 3 regions in the context of association to RFP. The important observation is that variation at polymorphic sites in these 3 regions is associated with RFP in both datasets. Evaluation of classification performances of models in validation tests (Table 2) indicates that both BNCs accurately captured interrelationships among all variables and are capable of predicting RFP classes from molecular data despite differences in structures of the BNC-IC and BNC-TOH.

BNCs showed equal accuracy of RFP-classification as LP-based models for both IC datasets in validation tests (Table 2). Meanwhile, only 5.0% decrease in classification accuracy was observed for BNC-TOH compared to LP-TOH. This finding indicates that, similar to LP models, BNCs are robust and HCV strains from patients with comparable yearly RFP have similar genetic properties extracted by both kinds of models. Because prediction performances of BNC models trained on randomized-labeled data deteriorate during validation tests, falling closely to the excepted classification accuracy of 50.0%, associations observed in data are not likely to be result of random correlations, thus, further providing evidence to support relevance of identified viral markers, accuracy of models performance and association to RFP.

Findings obtained from performance evaluations of LP and BNC models suggest that genetic heterogeneity at the identified polymorphic nt sites in the HCV core, NS3 and NS5b regions (Table 1) is relevant to RFP. This strongly implies that the genetic composition of HCV may influence liver disease progression. Circulation of potentially more aggressive HCV genotypes in human populations has been proposed to influence progression and severity of liver disease [18,39]. Moreover, the observation that different HCV genotypes and subtypes differ in responses to therapy [40] and epidemiology [5,13,16,21] provides further evidence of the potential impact that particular HCV strains may have on clinical outcomes in chronic hepatitis C.

## Conclusions

In conclusion, the Core, NS3 and NS5b genomic regions of the HCV 1b strains analyzed in this study were found to contain the RFP-relevant genetic markers, which were previously undetected by other analytical methods [18]. Here, for the first time, we show feasibility of developing robust and accurate genetic assays for the prediction of liver fibrosis progression in patients with chronic HCV infection using only HCV nt sequences. Development of such assays offers novel opportunities for clinical managements of patients and molecular surveillance of HCV-associated liver disease.

## Competing interests

Authors declare no competing interests.

## Authors' contributions

Conception and design: JL and YEK; data analysis: JL and YEK; data acquisition: FXLL, FGC and MB; manuscript drafting: JL, YEK, FXLL, FGC and MB.

## Declarations

Supported by Fondo de Investigación Sanitaria, Instituto de Salud Carlos III, Spanish Ministry of Economy Directorate of Science (Projects PI10/00512 and PI10/01734) and CIBER-ESP. FXLL holds a P.I. position supported by the Fondo de Investigación Sanitaria, Instituto de Salud Carlos III, Spain.

This article has been published as part of *BMC Bioinformatics *Volume 15 Supplement 8, 2014: Selected articles from the Third IEEE International Conference on Computational Advances in Bio and Medical Sciences (ICCABS 2013): Bioinformatics. The full contents of the supplement are available online at http://www.biomedcentral.com/bmcbioinformatics/supplements/15/S8.
